# Simulation-based teaching versus traditional small group teaching for first-year medical students among high and low scorers in respiratory physiology, India: a randomized controlled trial

**DOI:** 10.3352/jeehp.2025.22.8

**Published:** 2025-02-21

**Authors:** Nalini Yelahanka Channegowda, Dinker Ramanand Pai, Shivasakthy Manivasakan

**Affiliations:** 1 Institute of Health Professions Education, Sri Balaji Vidyapeeth (Deemed to be University) and Sri Manakula Vinayagar Medical College and Hospital, Puducherry, India; 2 Medical Simulation Centre, Sri Balaji Vidyapeeth (Deemed to be University), Puducherry, India; 3 Department of Prosthodontics, Indira Gandhi Institute of Dental Sciences, Sri Balaji Vidyapeeth (Deemed to be University), Puducherry, India; The Catholic University of Korea, Korea

**Keywords:** Simulation training, Medical education, Medical student, India, Randomized controlled trials

## Abstract

**Purpose:**

Although it is widely utilized in clinical subjects for skill training, using simulation-based education (SBE) for teaching basic science concepts to phase I medical students or pre-clinical students is limited. Simulation-based education/teaching is preferred in cardiovascular and respiratory physiology when compared to other systems because it is easy to recreate both the normal physiological component and alterations in the simulated environment, thus a promoting deep understanding of the core concepts.

**Methods:**

A block randomized study was conducted among 107 phase 1 (first-year) medical undergraduate students at a Deemed to be University in India. Group A received SBE and Group B traditional small group teaching. The effectiveness of the teaching intervention was assessed using pre- and post-tests. Student feedback was obtained through a self administered structured questionnaire via an anonymous online survey and by in-depth interview.

**Results:**

The intervention group showed a statistically significant improvement in post-test scores compared to the control group. A sub-analysis revealed that high scorers performed better than low scorers in both groups, but the knowledge gain among low scorers was more significant in the intervention group.

**Conclusion:**

This teaching strategy offers a valuable supplement to traditional methods, fostering a deeper comprehension of clinical concepts from the outset of medical training.

## Graphical abstract

[Fig f4-jeehp-22-08]

## Introduction

### Background/rationale

From the academic year 2019–2020 onward, medical education in India introduced a new curriculum known as competency-based medical education. In accordance with the National Medical Commission-prescribed curriculum, first-year MBBS (Bachelor of Medicine, Bachelor of Surgery) students are exposed to various teaching methods, including didactic lectures, small group teaching (SGT), demonstrations, directly observed procedural skills, early clinical exposure, and attitude and communication. Currently, the core undergraduate curriculum includes competencies such as communication skills, history-taking, attitudes and ethics in healthcare, physical examination, procedural and diagnostic skills, resuscitation skills, critical thinking, and problem-solving [1].

Physiology is one of the basic science subjects and is considered a creative, constructivist discipline that benefits from discussion and debate through student–teacher and student-to-student interactions. Small group teaching is defined as an educational technique in which learners exhibit 3 common characteristics: active participation, engagement in a specific task, and reflection. The advantage of group work is that it facilitates deep, active, and collaborative learning. In contrast to traditional lectures, collaborative testing engages students through discussion, providing the emotional and intellectual support necessary to extend their knowledge and achieve shared goals. However, small group work can be challenging for both students and faculty [2]. Faculty members often find the execution of SGT difficult because of large group sizes and limited faculty resources. Typically, each batch in an MBBS program comprises between 100 and 250 students; in our institute, there are 250 students per year. The topics chosen for SGT are clinically relevant “must-know” areas, and the current method—PowerPoint-based teaching followed by textbook discussions and a Q&A session—presents challenges such as a high student–teacher ratio, limited individual attention, and passive learning.

Simulation-based learning is a powerful educational tool that can be tailored to benefit both high and low scorers. By providing interactive, experiential learning opportunities, simulations address a range of learning needs and help bridge the gap between theoretical knowledge and practical application.

Although simulation-based education (SBE) is widely used in clinical subjects for skill training, its use for teaching basic science concepts to Phase I (pre-clinical) medical students is limited [3]. SBE is preferred in cardiovascular and respiratory physiology because it is relatively easy to recreate both normal physiological conditions and pathological alterations in a simulated environment, thus promoting a deeper understanding of core concepts [4].

### Objectives

The aim of this simulation-based small group educational practice was to design a teaching intervention that would cater to both high and low scorers, providing an immersive and engaging learning environment for first-year medical students, and to compare its effectiveness with traditional small group teaching (TSGT).

## Methods

### Ethics statement

Ethical approval was obtained from the Institutional Ethics Committee (IEC) of Mahatma Gandhi Medical College and Research Institute (IEC approval no., MGMCRI/RAC/2021/02/IHEC/10). Signed informed consent was obtained from all participants, in accordance with the ethics committee’s recommendations. This study forms a component of a larger trial registered on September 13, 2022, with the Clinical Trials Registry–India (CTRI) (CTRI/2022/09/045453).

### Study design

This study employed block randomization and consisted of 2 groups (intervention and control) with pre- and post-test analyses, along with an in-depth interview.

### Setting

The study was conducted by the Department of Physiology, in collaboration with the Medical Simulation Center, among first-year medical students at a deemed university in southern India. It was carried out between June and December 2022. A brief orientation regarding the intervention and assessment tools was provided to the students, and the intervention was implemented after the students had been exposed to clinical examinations in their curriculum.

### Participants

A total of 226 Phase I (first-year) medical undergraduate students were enrolled after providing informed consent. Students who did not attend at least 3 out of 4 assessments during the first-year physiology course were excluded from the study.

#### Pre-intervention preparation

Step 1 (needs analysis): To decide on the topics for the intervention, faculty opinions were collected regarding respiratory physiology topics that required additional resources (e.g., models and videos). Based on the topics suggested by the faculty, student opinions were gathered via an online survey in which they rated the listed topics in terms of difficulty. One topic was chosen from the list based on feasibility and clinical relevance.

Step 2 (pilot study): A pilot study was conducted with a group of 30 students who were not part of the main study. Their feedback on the simulation-based intervention—focusing on the process, the model used, and the assessment tools and feedback questionnaire—was collected. Based on this pilot study, modifications were made to the feedback questionnaire and the duration of the simulation-based intervention.

### Interventions

#### Didactic lecture

All students were introduced to the respiratory topics of “work of breathing and lung compliance in obstructive and restrictive lung disorders” via a didactic lecture. Delivered in 2-hour sessions, the lecture covered topics such as surfactant, lung compliance, work of breathing, lung volumes and capacities, and spirometry findings in obstructive and restrictive lung disorders. The content was standard for a medical physiology course and based on the department’s learning objectives.

#### Pre-briefing session

Students received a 10-minute briefing that outlined the learning outcomes, provided a recap of key physiological concepts, and demonstrated working models via video. They were then divided into 3 groups of 20 each. In a simulation training session, facilitators discussed the objectives and content, demonstrating a standardized approach to ensure the same content was delivered across 3 groups.

The role of surfactant in reducing surface tension was demonstrated to students using an experiment involving soap water (artificial surfactant), a coin, wood, and a clip. With the help of a facilitator, 5 case histories along with pulmonary function tests on obstructive and restrictive disorders were discussed. Alterations in ventilation and breath sounds were demonstrated using a lung model. This intervention was designed to align with the clinical examination demonstration classes for first-year MBBS students, thereby facilitating case-based discussions.

### Set induction

**Fig. 1.**
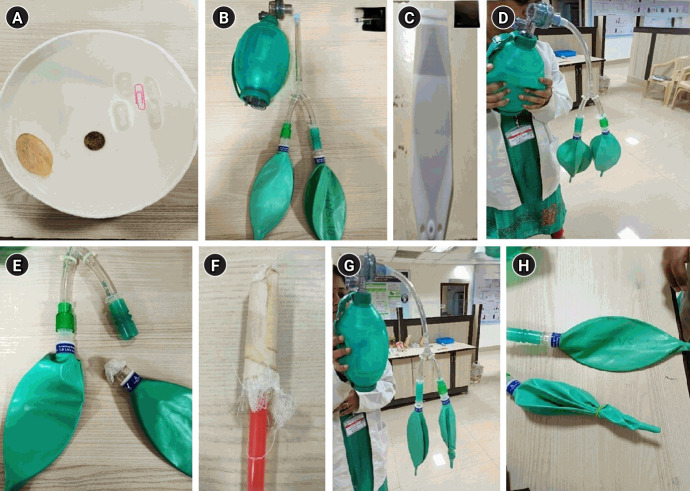
Description of the lung model. (A) Experiment to explain the role of surfactant in an alveoli (wood, coin, paper clip). (B) Working lung model consisting of bag and mask ventilation and 2 alveolar units. (C) Test lung to explain the concept of elastic recoil lung. (D) Obstructive lung disorder recreated using gauze in the airway (right alveolar unit). (E) Gauze piece blocking the airway similar to mucus in bronchial asthma. (F) Recreation of thickened airway similar to chronic bronchitis. (G) Recreation of restrictive lung disorder using rubber band (right alveolar unit). (H) Closer view of right alveolar unit.

A video demonstration on surface tension and the role of surfactant was shown using a bowl of water and objects of varied density (e.g., a coin, wood, and a paper clip). A soap solution was prepared, and a drop was placed on the paper clip to illustrate how surfactant reduces surface tension in the alveoli, thereby promoting better lung expansion (Fig. 1A).

### Description of the lung model

A lung model was constructed using 2 alveolar units, 3 plastic tubes of equal diameter and length, and a 3-way hose connector (Fig. 1B). Bag and mask ventilation was used to inflate and deflate the model. Prior to the demonstration, it was emphasized that the time required to fill 50% of both alveolar units with air is the same because (1) the tubes are identical in length and diameter (ensuring similar airflow resistance) and (2) the alveolar units have similar compliance, volume, and material [5]. Additionally, a test lung was used alongside the model to explain the role of the thoracic cavity in lung elastic recoil during expiration (Fig. 1C).

#### Recreation of the altered lung pathology using the model

Obstructive disorder: A gauze was used to recreate obstruction in the airway mimicking mucus secretion seen in obstructive lung disorders like bronchial asthma. It was then explained that expiration was altered, resulting in air trapping (Fig. 1D, E). The diameter of the airway was narrowed utilizing micropore gauze to simulate the thickened, narrowed airway observed in bronchitis. This reduced airway was inserted into one of the alveolar units to explain why bronchitis does not respond to bronchodilators, in contrast to bronchial asthma where patients experience immediate relief (Fig. 1F).

Restrictive disorder: A restrictive lung pattern was simulated by tying a rubber band around an alveolar unit to prevent air entry (Fig. 1G, H). This demonstration was complemented by discussions of case scenarios involving obstructive and restrictive lung disorders, along with spirometry and clinical examination findings (Supplements 1, 2).

### Control group

The control group was divided into groups of 20 with a facilitator and exposed to TSGT using PowerPoint presentations and textbook-based discussions. For the first 40 minutes, the facilitator discussed the salient points of the PowerPoint presentation—focusing on lung compliance, obstructive and restrictive lung disorders (including clinical and spirometry findings), and work of breathing. Students then engaged in textbook discussions, peer interaction, and doubt clarification with the facilitator. After data collection, the control group was subsequently exposed to the simulation-based teaching intervention.

### Outcomes

The study aimed to determine the effectiveness of the simulation-based intervention in terms of knowledge gain among first-year medical students and to compare its impact on low scorers versus high scorers.

### Measurements

We conducted an educational evaluation and obtained student feedback using a mixed-method approach that included both quantitative (questionnaire) and qualitative (group interviews) components.

#### Educational evaluation

The effectiveness of the teaching intervention was assessed using pre- and post-tests comprising 20 multiple-choice questions (MCQs), 6 extended matching items (EMIs), and 2 script concordance test (SCT) questions. These assessment tools were designed to test various levels of Bloom’s taxonomy—from basic recall to higher-level analysis and evaluation. MCQs primarily tested recall, EMIs assessed understanding, and SCTs evaluated analysis and evaluation [6]. Guidelines by Fournier et al. [7] were followed for SCT preparation. The assessment tools were validated by one external subject expert and 2 medical education experts.

#### Feedback survey (quantitative)

Of the 55 students who received the simulation-based intervention, 44 completed the survey. Student feedback regarding the teaching–learning intervention (TLI) was collected via a self-administered structured questionnaire using an anonymous online survey with a 5-point Likert scale ranging from strongly agree to strongly disagree. The questionnaire was prepared and validated in a pilot study involving 10 first-year medical students who were not part of the TLI. Based on their feedback, modifications were made to a few questions.

#### Procedure of the in-depth interviews

In-depth interviews were conducted by the first author in collaboration with a faculty member from the Department of Community Medicine, who is trained in qualitative interviewing. A convenience sampling approach ensured representation from both high- and low-scoring groups. Ten student volunteers were selected to explore their attitudes, behaviors, and perceptions regarding the simulation-based teaching intervention, with an emphasis on identifying both facilitating factors and shortcomings [8].

Interviews were scheduled at times convenient for both the students and the facilitator. A structured interview guide was prepared, and written informed consent was obtained from all participants. Each interview, which lasted approximately 50 minutes, involved discussing 8 guide questions. The discussions were documented by a scribe and digitally audio-recorded. Interviews continued until saturation of key concepts was reached. The digital audio-recorded interviews were transcribed verbatim, and the anonymized, pre-analysis text data were cross-verified with manually written transcripts by the scribe. The transcripts of the interviews were analyzed by 2 independent reviewers. Broad themes and important codes were generated from the transcripts and a common consensus was obtained between 2 reviewers with the help of the third reviewer. The themes and codes generated in the in-depth interview are summarized in Table 1. When some points were raised by a few participants and agreed upon by others—indicated by a thumbs-up gesture—these were noted.

### Bias

Selection bias was minimized through block randomization, and evaluator bias was reduced by using objective assessments and a default scoring system.

### Study size

This study employed block randomization and consisted of 2 groups (intervention and control) with pre- and post-test analyses, along with an in-depth interview. The sample size was calculated using G*Power 3.1 (Heinrich-Heine-Universität Düsseldorf), assuming a moderate to large effect size (Cohen’s d=0.68), 80% power, and α=0.05. The required sample size was 74 participants (37 per group), with an enrollment target of 120 (60 per group) to account for dropouts. A total of 107 students (intervention: 55, control: 52) completed the study and were included in the final analysis.

### Randomization:

**Fig. 2.**
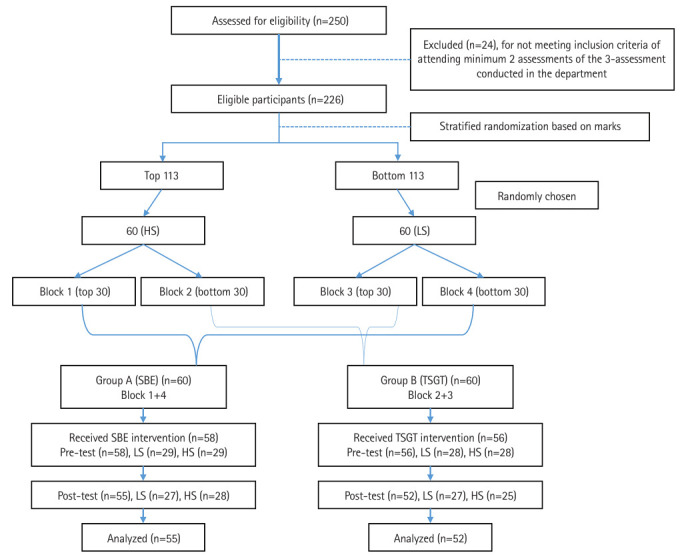
A modified CONSORT (Consolidated Standards of Reporting Trials) flow diagram for the individual randomized controlled trial of a teaching–learning intervention with the randomization process. SBE, simulation-based education; TSGT, traditional small group teaching; LS, low scorers; HS, high scorers.

All 250 Phase I (first-year) medical undergraduate students were initially enrolled after providing informed consent. The study was conducted between June and December 2022, and students who did not attend the required assessments during the first-year physiology course were excluded. The remaining 226 students from the academic year 2021–2022 were arranged in descending order based on their scores from departmental assessments (module and summative tests). From the top 113 students, 60 were randomly selected using an online random number generator and designated as high scorers; similarly, 60 were randomly selected from the bottom 113 and designated as low scorers. These 120 students were then divided into sequential blocks of 30. Blocks 1 and 4 were combined to form Group A (SBE), and Blocks 2 and 3 were combined to form Group B (TSGT), ensuring an equal number of high and low scorers in both groups. A coin toss was used to decide which group would receive the intervention. A modified CONSORT flow diagram for this randomized controlled trial is provided in Fig. 2. This study followed CONSORT guidelines where applicable, but certain modifications were made like lack of allocation concealment, blinding of assessors and absence of Intention to Treat analysis based on study design constraints.

### Blinding

Participants and assessors were not blinded for the intervention.

### Unit of analysis

The groups (control and intervention) served as analysis units.

### Statistical methods

Data were entered into a Microsoft Excel spreadsheet (Microsoft Corp.) and saved as a comma-separated values file. Frequency distributions of the teaching modalities were calculated. Scores were tested for normality using normal probability plots and the Shapiro-Wilk test. Paired sample t-tests were conducted to compare pre- and post-test scores, with a P-value of <0.05 considered statistically significant. The raw response data and cumulative scores for the intervention and control groups are available as Datasets 1, 2, and 3, respectively.

## Results

The post-test scores of the intervention group were significantly higher than the pre-test scores, whereas the control group’s post-test scores, although higher than their pre-test scores, did not show a statistically significant difference (Table 2). A comparison of knowledge gain (post-test minus pre-test scores) between the intervention and control groups revealed a statistically significant difference using an independent t-test (Table 3).

Sub-analysis comparing high and low scorers within both groups showed that high scorers performed significantly better than low scorers in both the intervention and control groups (Table 4). Moreover, the knowledge gain among low scorers in the intervention group was significantly greater than that among low scorers in the control group (Table 5). To capture students’ perceptions of the TLI, a satisfaction index was calculated using the following formula for questions 2, 7, 8, 9, and 12 [9]:

Satisfaction index

n1*1+n2*2+n3*3+n4*4+n5*5*100

N*5

**Fig. 3.**
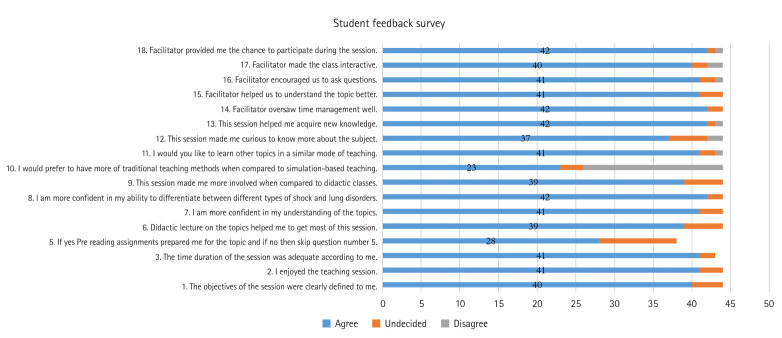
Student feedback survey on simulation-based education.

In the above formula n1, n2, n3, n4, and n5 are the number of students who marked the responses 1, 2, 3, 4, and 5 on the Likert scale, respectively, and N is the total number of students. Overall, 93.6% of the students enjoyed the session, 88.6% felt more confident in their understanding of the topics and in their ability to differentiate between obstructive and restrictive lung disorders, and 89% reported that the session helped them acquire new knowledge. Approximately 86% stated that the session made them more involved and curious (Fig. 3).

## Discussion

### Key results

This investigation demonstrated that first-year medical students who received a simulation-based teaching intervention scored significantly higher than those exposed to TSGT. Notably, simulation-based teaching was particularly effective among low scorers.

### Interpretation

This study examined simulation as a supplementary teaching strategy alongside conventional methods. The data support the hypothesis that first-year medical students learn respiratory physiology concepts more effectively when lectures are paired with simulation—thus introducing clinical skills earlier in the curriculum rather than during the later years (second to fourth year). A similar observation was made in a study using the Harvey simulator after lectures on cardiovascular physiology, which led to significant knowledge gains in first-year students [10].

There is also evidence that case-based teaching enhances knowledge retention among basic science students compared to traditional lecture–laboratory settings. For example, Malau-Aduli et al. found that integrating case-based instruction led to enhanced retention and a higher perception of subject relevance [11].

### Comparison with previous studies

Similar to our findings—where students reported a better understanding of lung disorders—Dutt et al. [12] observed that an integrated simulation-based early clinical exposure module in cardiovascular physiology improved communication (93.8%) and clinical skills, as well as participation and understanding of patient care (99.3%) among first-year students.

Providing diverse learning experiences is crucial. Studies have highlighted that factors such as proper syllabus planning, prior information about assessments, and clearly stated learning objectives promote focused learning.

Our teaching strategy combined multiple learning modalities—reading (pre-reading assignments), visual (PowerPoint, videos, case scenarios), auditory (videos, discussions), and kinesthetic (models of normal and altered lung pathology)—to address the varied learning preferences of our students. In our study, low scorers in the intervention group showed significantly greater improvement than low scorers in the control group. One low scorer noted during the in-depth interview that the visualization and step-by-step discussion greatly enhanced his focus, which is a notable strength of the intervention.

High scorers in both groups also improved, suggesting that intrinsically motivated students benefit regardless of the teaching method; however, the simulation-based approach appears especially beneficial for those who initially struggle [13].

In our study, 93.6% of the students enjoyed the session, and 88.6% felt more confident in their understanding of the topics. Similarly, a study by Suvarna and Basti [14] among first-year students in Karnataka reported that simulation exercises provided a realistic and valuable learning experience that improved understanding of physiology concepts.

The integration of simulation-based teaching as an adjunct modality for first-year students is an effective tool for understanding the pathophysiological basis of common clinical conditions such as obstructive and restrictive lung disorders—a key strength of this study [15].

### Limitations

Long-term retention of knowledge was not assessed. Moreover, multiple faculty members with distinct teaching styles and approaches were involved, which may have introduced variability in the intervention.

### Generalizability

Because a random sampling method was employed, the study findings may be generalized to pre-clinical medical education among first-year students. However, potential confounding factors—such as student gender, performance expectations, and teacher-related variables (e.g., multiple facilitators and scheduling issues)—should be carefully considered when extrapolating these results to larger populations.

### Suggestions

A longitudinal study following the same cohort through their first, second, and final years of the MBBS course and internship is recommended.

### Conclusions

Simulation-based teaching was more effective than TSGT for both high and low scorers. This enhanced effectiveness is likely due to factors such as the real-time application of learned content, active participation, and immediate feedback.

## Figures and Tables

**Figure f4-jeehp-22-08:**
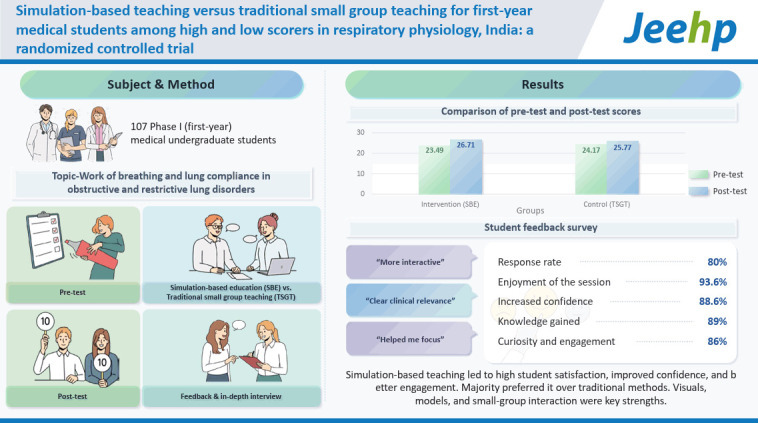


**Table 1.** Outcomes of the in-depth interviews

Serial no.	Themes	Codes
1	Teaching–learning intervention	Topics (9), Clinical relevance (7), Case based scenarios (19), Models (7), Visualization (3), Clear (10), Knowledge application (2), Sequence (2), Demonstration (7)
	1) What do you think of this simulation-based teaching–learning intervention?	**Transcript**
	2) Was this teaching intervention any different from what you have been exposed to until now?	“I would like to add that, so the clinical relevance we got the relevance of the content while you are learning something and it was very organized. So that made it more interesting!”
		“In my case, it was what we were seeing. It gave us clear-cut idea of how to approach a patient also in different aspects like step by step, how to approach a patient and then what are the clinical core relations with them if this patient is appearing. So, it brought more focus.”
		“I would like to add that previously I asked you a question also regarding the class—can I write on the student corners regarding COPD and compliance? So, I was struggling through that because I did have lots of pages in Google and I couldn’t find it out”
		“The case study also helps us differentiate. And how can you differentiate it? How do we compare it to more hands-on and more understandable than the text”
3	How was your facilitator’s interaction during this teaching–learning intervention?	Feedback (5), Small group (4), Interaction (8), Individual attention (2), Discussion (13), Video (11)
		**Transcript**
		“It actually made clear to us the physiological basis of the things happening. If it’s OLD or if it’s RLD, how it’s happening. That model was very explicit.”
		“Another thing and I think also encouraged other groups to input their opinions of the other people’s tables to understand what we understand and what are possible opinions people can reach for a particular case? Another person could be this, might be another so that penetrates a more interpersonal interaction between us as a team.”
		“Instant clearing of doubts”
		“Comfortable learning environment”
4	What do you think of the pre-reading assignments?	Stimulus (4), Instructions (3), History taking (2)
		**Transcript**
		“For me, a video was presented before the actual intervention that helped me a lot in understanding what is expected of me.”
		“Also, I want to say that the pre-reading assignments and you constantly telling us that you are going to do something in the coming days kept us curious and eager”
5	What do you think of the assessments?	Unfamiliar (2), Curiosity (3)
6	1) Can you comment on the timing of the intervention? Time duration	Not sufficient (5), Overlap with other (2)
	2) Is first year too early to introduce simulation-based teaching of clinically relevant topics?	
	3) Did you face any difficulty in understanding any findings?	

COPD, chronic obstructive pulmonary disease; OLD, obstructive lung disease; RLD, restrictive lung disease.

**Table 2.** Mean pre-test and post-test scores in the intervention group and control group

Group	Pre-test	Post-test	t-test (P-value)
Intervention (n=55)	23.49±6.62	26.71±7.83	–2.64 (0.01^*^)
Control (n=52)	24.17±6.21	25.77±6.51	–1.48 (0.14)

Values are presented as mean±standard deviation.

* P<0.05 is statistically significant and indicated in boldface; by paired t-test.

**Table 3.** Comparison of knowledge gain (post-test minus pre-test scores) between the intervention and control groups

Groups	Pre-test	Post-test	Post test–pre test	t-test (P-value)
Intervention	23.49±6.62	26.71±7.83	3.22±1.2	9.38 (0.01^*^)
Control	24.17±6.21	25.77±6.51	1.60±0.3	

Values are presented as mean±standard deviation.

* P<0.05 is statistically significant and indicated in boldface; by independent t-test.

**Table 4.** Mean pre-test and post-test scores of high and low scorers in the intervention group and control group

	Pre-test	Post-test	t-test (P-value)
Intervention group (n=55)			
High scorers (28) (A1)	21.57±4.32	24.39±5.38	2.48 (0.009^*^)
Low scorers (27) (B2)	17.4±4.46	19.4±5.44	1.48 (0.074)
Control group (n=52)			
High scorers (25) (A2)	20.07±4.75	23.19±4.91	3.10 (0.002^*^)
Low scorers (27) (B2)	20.92±4.42	19.6±4.12	–1.118 (0.274)

Values are presented as mean±standard deviation.

* P<0.05 is statistically significant and indicated in boldface; by paired t-test.

**Table 5.** Comparison of knowledge gain (post-test minus pre-test scores) of high and low scorers between the control and intervention groups

	Pre-test	Post-test	Knowledge gain t-test (P-value)
Intervention group (n=55)			
High scorers (28) (A1)	21.57±4.32	24.39±5.38	B1–B2
Low scorers (27) (B1)	17.4±4.46	19.4±5.44	–1.845 (0.035^*^)
Control group (n=52)			
High scorers (25) (A2)	20.07±4.75	23.19±4.91	A1–A2
Low scorers (27) (B2)	20.92±4.42	19.6±4.12	0.19106 (0.42)

Values are presented as mean±standard deviation.

* P<0.05 is statistically significant and indicated in boldface; by independent t-test.

## Footnotes

**Data availability**
Data files are available from Harvard Dataverse: https://doi.org/10.7910/DVN/IJER4V Dataset 1. Raw responses data and cumulative scores from participants. (intervention group).

Dataset 2. Raw responses data and cumulative scores from participants. (control group).

Dataset 3. Raw responses data from the student feedback.

## Supplementary materials

Supplementary files are available from Harvard Dataverse: https://doi.org/10.7910/DVN/IJER4V

Supplement 1. Clinical scenario: Bronchial asthma.

Supplement 2. Clinical Case History: Pleural effusion secondary to carcinoma of the breast.
