# Supplementary material for: Simulation-based teaching versus traditional small group teaching for first-year medical students among high and low scorers in respiratory physiology, India: a randomized controlled trial
Source: J Educ Eval Health Prof. 2025 Feb 21;22:8. doi: 10.3352/jeehp.2025.22.8 (PMC12012709; doi:10.3352/jeehp.2025.22.8)
Supplement: Supplementary file 4 — Supplement 1. Clinical scenario: Bronchial asthma. [file jeehp-22-08-suppl1.docx]

**Clinical scenario: Bronchial asthma**

A 60 years old male patient came to pulmonary medicine OPD with chief complaints of breathlessness, and cough. He had frequent episodes of allergic rhinitis symptoms such as runny, blocked, itchy nose and snoring at sleep. The patient was diagnosed with mild persistent asthma and allergic rhinitis since the age of 6 years. By at age of 12, the asthma symptoms almost disappeared except some of cough and wheezing when he got flu. He is a nonsmoker and is on regular inhaled bronchodilators and inhaled corticosteroids for the past three years.

On examination,

Pulse - 120 bpm

Respiratory rate - 20/min.

Blood pressure - 120/80 mm Hg

SpO2: 98% on room air

On Auscultation, there was bilateral expiratory wheeze with vesicular breath sounds. There were no other abnormal findings on examination.

**Investigations:**

**Chest X ray was normal**

**Spirometry**

|  |  | Predicted |
| --- | --- | --- |
| FEV_1_ (L) | 3.2 | 3.6 – 4.2 |
| FVC (L) | 5.0 | 4.5 – 5.4 |
| FEV_1_/FVC (Pre) | 64 % | 75 - 80 |
| FEV_1_/FVC (Post Bronchodilator) | 78 % | 75-80 |
| PEF (L/min) | 400 | 440 - 540 |

Reference - Medical Council of India. Early Clinical Exposure for the Undergraduate Medical Education Training Program, 2019: pp 1-43.
