# Supplementary material for: Simulation-based teaching versus traditional small group teaching for first-year medical students among high and low scorers in respiratory physiology, India: a randomized controlled trial
Source: J Educ Eval Health Prof. 2025 Feb 21;22:8. doi: 10.3352/jeehp.2025.22.8 (PMC12012709; doi:10.3352/jeehp.2025.22.8)
Supplement: Supplementary file 5 — Supplement 2. Clinical Case History: Pleural effusion secondary to carcinoma of the breast. [file jeehp-22-08-suppl2.docx]

**Clinical Case History: Pleural effusion secondary to carcinoma breast**

**Case history**

A 57-year-old woman with presents with complaints of fever, non-productive cough, and worsening shortness of breath of 2 days duration. Woman also complains loss of appetite and weight loss for 3 months. The woman gives a history of lump in the right breast for three months.

**On examination,**

Vitals

Blood pressure 123/56 mmHg

Pulse 88 beats per min

Respiratory rate 36 per min

Temperature 101.3 F

Oxygen saturation 96% on room air.

Trachea is shifted to left

Auscultatory findings

On percussion dull note is heard on the right auscultatory areas. (mammary and infra mammary regions)

Decrease of respiratory sounds in the right side.

**Investigations:**

**Chest X ray – mediastinal shift noted and obliteration of Costo-phrenic angle.**

**Spirometry**

|  |  | Predicted |
| --- | --- | --- |
| FEV_1_ (L) | 3.2 | 3.6 – 4.2 |
| FVC (L) | 4.2 | 4.5 – 5.4 |
| FEV_1_/FVC | 76 | 75 - 80 |
| PEF (L/min) | 460 | 440 - 540 |
